# Volumetric Modulated Arc Therapy for lung cancer: Correlation between dose and volume

**DOI:** 10.1002/acm2.70781

**Published:** 2026-09-23

**Authors:** Yu Wang, Deying Xu, Yunfeng Liu, Xiaofeng Zhang, Liang Yu

**Affiliations:** ^1^ Radiotherapy Center Panjin Central Hospital Panjin Liaoning Province China; ^2^ Department of Radiotherapy Kangbao County People's Hospital Zhangjiakou Hebei Province China

**Keywords:** linear accelerators, lung cancer, mean lung dose, radiation therapy, volume ratio

## Abstract

**Background:**

In lung cancer radiotherapy, the volume of the lung receiving a dose above 20 Gy (V_20Gy_) and the mean lung dose are widely recognized dosimetric indicators for predicting radiation pneumonitis. However, there is currently no unified model to predict the minimum achievable values of V_20Gy_ and mean lung dose. In modern radiotherapy, the most advanced and widely used technology in mainstream medical linear accelerators is volumetric modulated arc therapy (VMAT). This article describes, based on linear accelerator VMAT, the clinical dependency of achievable dose‐to‐volume ratios.

**Objective:**

To investigate the predictive effect of the ratio of the planning target volume (PTV) to lung volume (r) on lung V_20Gy_ and mean lung dose (MLD) in volumetric modulated arc therapy (VMAT) for lung cancer, with the aim of optimizing radiotherapy plans for lung cancer.

**Methods:**

A total of 197 samples were used, including VMAT plans covering primary lung lesions and lymphatic drainage areas, of which 149 were used for training and 48 for testing. A retrospective analysis was conducted on the relationship between the r and MLD or V_20Gy_ in the plans, and function fitting was performed using the least squares method.

**Results:**

The r ranged from 0.0112 to 0.2811. The R^2^ values of the power function and logarithmic function of MLD versus r were 0.820 and 0.805, respectively, while the *R* ^2^ values of the power function and logarithmic function of V_20Gy_ versus r were 0.822 and 0.816, respectively. The residual of MLD is ± 200, and the residual of V_20Gy_ is ± 4%, with residuals distributed randomly.

**Conclusion:**

The r showed good fitting results with the power and logarithmic functions of MLD or V_20Gy_. During treatment plan creation and evaluation, the power or logarithmic function relationships can be used to predict MLD and V_20Gy_ in the treatment plan, thereby achieving the lowest possible lung dose while conforming to radiation principles.

## 1 INTRODUCTION

In the field of radiotherapy for lung cancer, stereotactic radiosurgery (SRS) and stereotactic body radiotherapy (SBRT) achieve higher local control rates and lower toxicity for primary tumors, oligometastases, and peripheral lung cancer.^1^, ^2^ However, for radiotherapy covering the primary tumor, hilar, and mediastinal lymphatic drainage areas, intensity‐modulated radiotherapy with conventional fractionation is more appropriate. Although radiotherapy is effective in local control of lung cancer, its associated toxic side effects should not be underestimated. Radiation‐induced lung injury (RILI) that occurs after thoracic radiotherapy mainly manifests as radiation pneumonitis (RP),^3^, ^4^, ^5^, ^6^, ^7^, ^8^, ^9^ and the occurrence of radiation pneumonitis can to some extent reduce the value of radiotherapy for lung cancer.

In chest tumor radiotherapy,^10^, ^11^, ^12^ the main indicators for lung dose limitation include: The percentage of lung volume receiving more than 20 Gy (V_20Gy_), the percentage of lung volume receiving more than 5 Gy (V_5Gy_), and the mean lung dose (MLD) of both lungs (MLD). The MLD and lung V_20Gy_ are widely recognized as two indicators used to predict radiation pneumonitis.^13^, ^14^, ^15^ Therefore, it is particularly important to strictly limit MLD and V_20Gy_ when formulating a radiotherapy plan. A high‐quality treatment plan should aim to minimize the lung dose as much as possible while ensuring the target volume receives the prescribed dose, in order to achieve the maximum therapeutic benefit. Additionally, doses may vary for plans generated by different treatment centers or different linear accelerators and planning systems, so an indicator is needed to predict minimum achievable levels of MLD and V_20Gy_ reasonably. The ratio of PTV volume to lung volume (r) has been less mentioned in previous studies; only a few studies have indicated that GTV or PTV size is related to the incidence of radiation pneumonitis.^16^ However, lung volumes differ significantly among patients; for example, in this study, the all lung volume ranged from 1741.96 mL to 5687.99 mL. Therefore, compared to GTV or PTV volume, the r may be a more valuable factor for predicting lung dose.

In modern radiotherapy technology, medical linear accelerators are the most widely used and efficient radiotherapy devices. Among them, volumetric modulated arc therapy (VMAT) with linear accelerators offers good target conformity and relatively short treatment times. Therefore, this paper focuses on studying the dose received by the lungs under coplanar dual‐arc VMAT treatment plans.

Since peripheral lung cancer often uses SBRT, this study only retrospectively analyzes treatment plans for patients with central lung cancer who received radical radiotherapy. The aim is to explore how the MLD or V_20Gy_ changes with r, investigate the relationship between lung dose and regions of interest (ROIs) like the planning target volume (PTV) and lung volume, and ultimately help design more precise radiotherapy plans.

## 2 MATERIALS AND METHODS

### 2.1 Patient selection and planning criteria

This study collected a total of 197 central lung cancer cases treated with volumetric modulated arc therapy (VMAT) from the past five years (2021 to 2025) across two centers for a retrospective analysis. Among them, 149 cases from Center 1 were used as the training set, and 48 cases from Center 2 were used as the test set, with a prescribed dose of 60 Gy/30F. Inclusion criteria were: Age over 18, no restriction on gender, and meeting the indications for radiotherapy. The target volume included the primary lung lesion and the hilar or mediastinal lymphatic drainage regions. Centrally located lung cancer was defined according to the RTOG0915 report^17^ (central if within 2 cm of the proximal bronchial tree, peripheral if more than 2 cm). All plans used coplanar VMAT plans with dual‐arc. CT simulation positioning was done using the uRT‐linac506C integrated CT linear accelerator from United Imaging Healthcare (UIH) for standard 3D CT positioning. The expansion of safety margins were determined comprehensively based on factors such as the patient's pathological stage and type、age、and physical condition.

The prescribed dose of the plan is required to cover more than 95% of the PTV volume, and the plan is ultimately normalized to V_60Gy _= 95%, D_95% _= 60 Gy. Reference values for organ‐at‐risk(OAR) limits are based on the application of normal tissue complication probability models in clinical practice (QUANTEC).^18^ The incidence of symptomatic pneumonitis is related to various dose parameters and has no clear threshold, only statistical probability; when the mean dose to both lungs is less than 13 Gy, the probability of developing symptomatic pneumonitis is less than 10%; when MLD is less than 7 Gy, the probability of symptomatic pneumonitis is less than 5%; when V_20Gy_ of both lungs is less than 30%, the probability of symptomatic pneumonitis is less than 20%. When the mean dose to the heart is less than 26 Gy, the incidence of pericarditis is less than 15%. When the maximum dose to the spinal cord reaches 50 Gy, the probability of myelopathy is 0.2%. The maximum dose to the spinal cord should not exceed 45 Gy, and should be lowered as much as possible. The spinal cord is usually expanded by 0.3 cm to create a Planning Risk Volume (PRV) and is limited to below 45 Gy.

### 2.2 VMAT plan and treatment delivery

The treatment plan was designed using the uRT‐TPOIS planning system for the uRT‐linac 506C integrated CT linear accelerator from United Imaging Healthcare (UIH) in Shanghai. A 6 MV flattened (FF) beam mode was used, with a dose rate of 400 MU/min. The multileaf collimator (MLC) consists of 60 pairs of alloy leaves, with the middle 40 pairs having a width (equivalent to the central projection width) of 0.5 cm per leaf, and the side leaves having a width of 1 cm, forming a maximum irradiation area of 40 cm × 40 cm. All calculations were performed using the Monte Carlo algorithm, with a dose grid of 2.5 mm. Each plan included two 360° full arcs, irradiated clockwise and counterclockwise, respectively. A segment was created every 2°, resulting in 180 segment per arc. In each plan, the collimator angle for the two arcs were 10° and 350°, respectively. For the optimization constraints of all plans, the PTV weight was set to 70, and the weight for organs at risk was 10 or 20. Specific optimization constraint values were adjusted according to the above organ‐at‐risk reference guidelines and the characteristics of each case, ultimately normalized to cover 95% of the PTV volume with the prescription dose.

### 2.3 Fitting optimization

The r values, MLD, and V_20Gy_ of all samples were recorded. Lung volume refers to all lung volume, specifically the bilateral lung volume without subtracting the PTV. The curve fitting was performed using Matlab 2023b, with the fitting functions being linear, exponential, logarithmic, and power functions, corresponding to Equations (1), (2), (3), (4). Here, Y represents MLD or V_20Gy_, r represents the ratio of PTV to all lung volume, and A, B, and C are the coefficients to be determined in the fitting functions. The fitting functions are linear, exponential, logarithmic, and power functions, corresponding to formulas 1 through 4. Here, Y represents MLD or V_20Gy_, r represents the ratio of PTV to total lung volume, and A, B, and C are the coefficients to be determined in the fitting functions.

(1) Y=A×r+B

(2) Y=A×exp(B×r)

(3) Y=A+B×log(r)

(4) $$Y=A\times r^{B}+C$$

## 3 RESULTS

**FIGURE 1:**
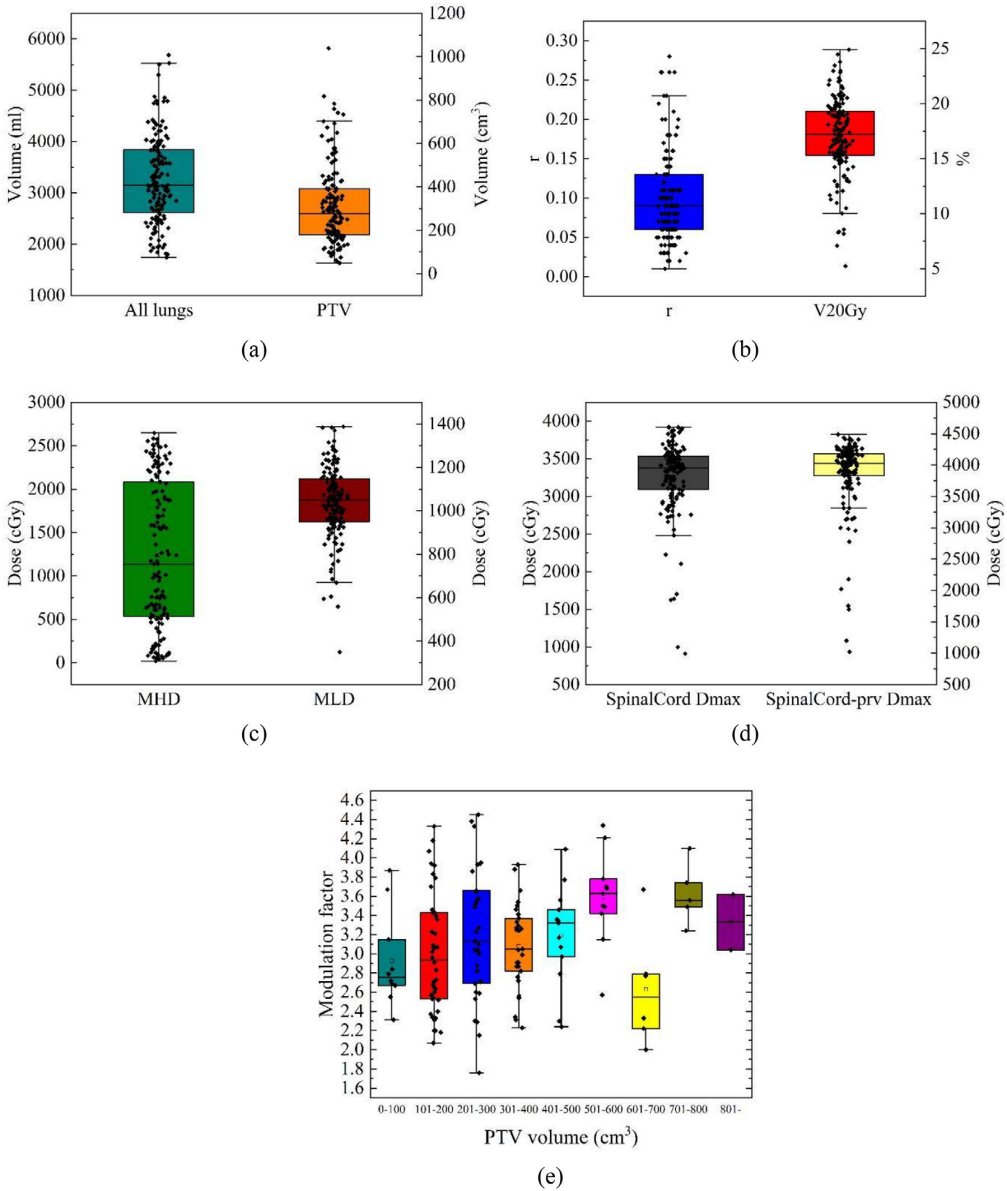
Statistical values of each parameter in the training set. (a). All lung volume and PTV volume; (b). The r and V_20Gy_ of lungs; (c). Mean heart dose (MHD) and mean lung dose (MLD); (d). Maximum dose of the spinal cord and spinal cord‐planning risk volume (prv); (e). Modulation factors of PTV volume groups in the training set.

**FIGURE 2:**
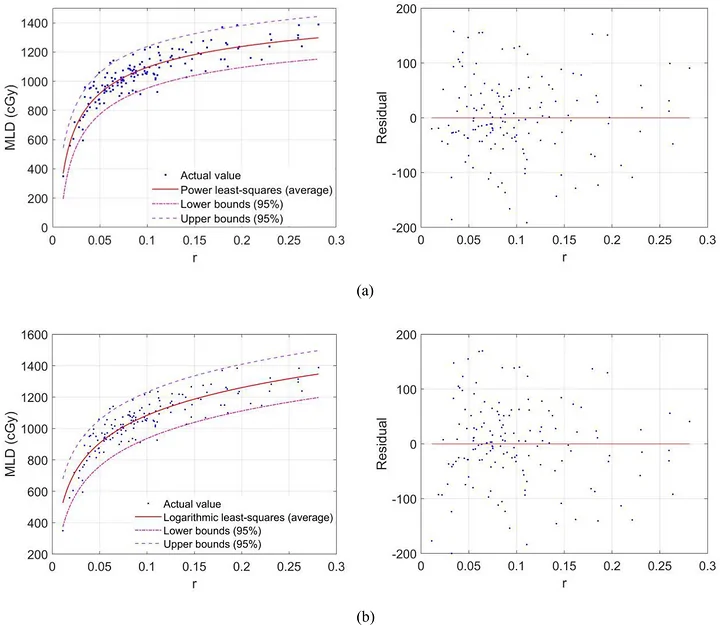
Fitting function and residual plot of MLD. (a). Power function fitting and residual plot of MLD; (b). Logarithmic function fit of MLD and residual plot. Captions: MLD: mean lung dose. The fitted curve chart includes: least squares fitted curve (average trend) and 95% upper and lower bounds.

Table 1 presents the statistical results of the volumes of regions of interest, lung dose, heart dose, and spinal cord dose in lung cancer treatment plans of the training set. Among them, in this study, there was one case with mean heart dose exceeding 26 Gy, and four cases with MLD exceeding 13 Gy. Table 2 shows the statistical results of the modulation factors grouped by PTV volume. Figure 1 shows the statistical charts of all lung volume, PTV volume, r, V_20Gy_, mean heart dose and MLD, and maximum spinal cord dose. Figure 2 is the statistical chart of modulation factors grouped by PTV volume.

**TABLE 1:** Volume and dose information of the region of interest.

Regions of interest (ROI)	Dose constraint	median	min	max
PTV (cm^3^)	—	276.32	49.11	1038.68
r	—	0.0851	0.01	0.28
Lungs	MLD < 13 Gy	10.50	3.49	13.88
	V_20Gy_ < 25%	17.21	5.24	24.91
	Volume (ml)	3145.95	1741.96	5687.99
Heart	Dmean(Gy) < 26 Gy	11.37	0.19	26.46
Spinal Cord	Dmax(Gy) < 40 Gy	33.76	9.11	39.18
Spinal Cord‐prv	Dmax(Gy) < 45 Gy	40.30	10.20	44.89

**TABLE 2:** Modulation factor for PTV volume grouping.

PTV (cc)	number of patients	median	min	max
0–100	10	2.75	2.31	3.87
101–200	42	2.93	2.07	4.33
201–300	31	3.13	1.76	4.45
301–400	29	3.05	2.23	3.93
401–500	13	3.32	2.24	4.09
501–600	11	3.63	2.57	4.34
601–700	6	2.55	2.00	3.67
701–800	5	3.56	3.24	4.10
800‐	2	3.33	3.04	3.62

Tables 3 and 4 show the statistical results of the fitting parameters for the MLD and V_20Gy_ in the training set, including the least squares fit of the average trend and the 95% upper and lower bound curves. Among them, the power and logarithmic functions have higher coefficients of determination (*R* ^2^) and smaller root mean square errors (RMSE) and sums of squared errors (SSE). Table 5 presents the goodness‐of‐fit results for MLD and V_20Gy_ in the test set. Similarly, the RMSE and SSE of the power and logarithmic functions are smaller than those of the linear and exponential functions. Therefore, this study uses the power and logarithmic functions for the function fitting of lung doses.

**TABLE 3:** Parameter statistics of the fitting function for the mean lungs dose (MLD) in the training set.

Function	Range	$R^{2}$	A	B	C	RMSE	SSE
Linear	Average trend (least‐squares)	0.646	2344	807.56	—	100.22	1476600
	95% Lower bounds		2061	775.18	—		
	95% Upper bounds		2626	839.93	—		
Exponential	Average trend (least‐squares)	0.607	849.41	1.9779	—	105.67	1641400
	95% Lower bounds		821.38	1.73	—		
	95% Upper bounds		877.43	2.23	—		
Logarithmic	Average trend (least‐squares)	0.805	1670	254.69	—	74.40	813710
	95% Lower bounds		1618	234.26	—		
	95% Upper bounds		1722	275.13	—		
Power	Average trend (least‐squares)	0.820	−352.48	−0.3155	1823	71.69	750270
	95% Lower bounds		−693.96	−0.4786	1393		
	95% Upper bounds		−10.99	−0.1524	2253		

**TABLE 4:** Parameter statistics of the fitting function for V_20Gy_ in the training set.

Function	Range	$R^{2}$	A	B	C	RMSE	SSE
Linear	Average trend (least‐squares)	0.671	49.70	12.10	—	2.01	594.86
	95% Lower bounds		44.02	11.45	—		
	95% Upper bounds		55.37	12.75	—		
Exponential	Average trend (least‐squares)	0.620	13.19	2.477	—	2.16	686.67
	95% Lower bounds		12.64	2.17	—		
	95% Upper bounds		13.73	2.78	—		
Logarithmic	Average trend (least‐squares)	0.816	30.23	5.336	—	1.5047	332.8121
	95% Lower bounds		29.18	4.92	—		
	95% Upper bounds		31.28	5.75	—		
Power	Average trend (least‐squares)	0.822	−16.77	−0.1927	44.23	1.4852	322.0532
	95% Lower bounds		−38.90	−0.36	19.90		
	95% Upper bounds		5.36	−0.03	68.56		

**TABLE 5:** The goodness of fit of the test set MLD and V20Gy.

	MLD		V_20Gy_	
Function	RMSE	SSE	RMSE	SSE
Linear	96.66	429790	2.30	243.28
Exponential	99.45	454980	2.49	285.88
Logarithmic	79.37	289790	1.64	124.11
Power	78.57	277830	1.63	119.53

**FIGURE 3:**
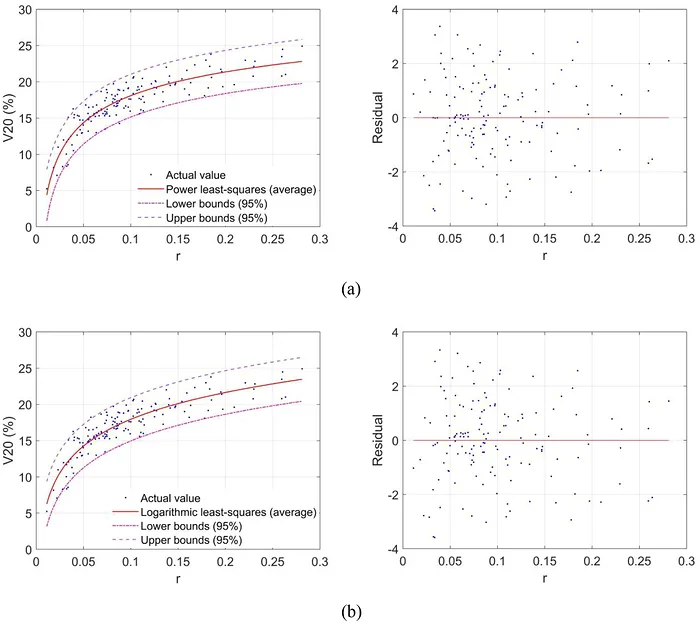
Fitting function and residual plot of V_20Gy_. (a). Power function fitting and residual plot of V_20Gy_; (b). Logarithmic function fitting and residual plot of V_20Gy_. Captions: The fitted curve chart includes: least squares fitted curve (average trend) and 95% upper and lower bounds.

The curve showing the fitted relationship between the MLD and r is shown in Figure 2. Panel (a) shows the fitted curve of the power function and its residual plot; panel (b) shows the fitted curve of the logarithmic function and its residual plot. This figure includes the least‐squares function of the average trend and the function fitting curves of the 95% upper and lower bounds. In addition, the residuals of both the power function and logarithmic function are randomly distributed at all stages. The curve and residual plot of the functional relationship between V_20Gy_ and r are shown in Figure 3. Similarly, it includes the least‐squares function of the average trend and the fitting curves of the 95% upper and lower bounds, where (a) is the fitted curve of the power function and its residual plot; (b) is the fitted curve of the logarithmic function and its residual plot, and the residuals are also randomly distributed.

## 4 DISCUSSION

By using tumor size、all lung volume, and their ratio to predict lung dose in advance, it can guide multidisciplinary consultations and discussions, and dosimetrists can estimate approximate numbers for MLD and V_20Gy_ in the optimization constraints just by using the PTV and all lung volume, saving time in the design of the treatment plan. For more advanced algorithms, like knowledge‐based planning (KBP),^19^ they use statistical models or machine learning methods to extract prior knowledge of dose distributions from historical cases and predict the optimal dose distribution for new patients. This can generate standardized, high‐quality radiotherapy plans and might have better predictive ability because it considers the geometric relationship between the PTV and organs at risk. However, advanced algorithms like KBP are routinely used by Eclipse users in the US, but they are not yet widely implemented in developing countries and regions. This paper provides a general trend, and specific factors such as the geometry and location of the target volume, beam arrangements, and other planning‐related variables are largely accounted for within a 95% confidence boundary. Therefore, this simple and efficient method in this study might be more suitable for clinical use in treatment centers that have not purchased or equipped KBP and could better aid in treatment planning and quality control of lung doses.

The study by Kabolizadeh et al. showed that there is a slight but significant difference between the two methods used to evaluate lung dose, namely all lung minus PTV and all lung minus GTV.^20^ This difference should be considered when comparing dosimetric information and optimizing treatment plans between different institutions. Wang et al.’s study indicated that the MLD and V_20Gy_ vary greatly depending on different lung definition methods, and the MLD calculated by excluding the GTV may more accurately predict clinically significant radiation pneumonitis.^21^ Tomoko et al.’s study analyzed risk factors for symptomatic RP after lung cancer SBRT and found that the ratio of PTV to lung volume is a good predictor of symptomatic RP after SBRT.^22^ Hoffman et al., through a multicenter retrospective analysis, studied the correlation of gradient index (GI) and R50% with PTV volume in lung cancer SBRT. Using the least squares method to fit GI and R50% metrics with a power function, they found good fitting results for PTV volumes greater than 85 cm^3^.^23^ Vinogradskiy et al., by predicting radiation pneumonitis models, investigated the effect of tumor location on radiation pneumonitis and found that the incidence of upper lung radiation pneumonitis was lower, though it did not reach statistical significance.^24^

**FIGURE 4:**
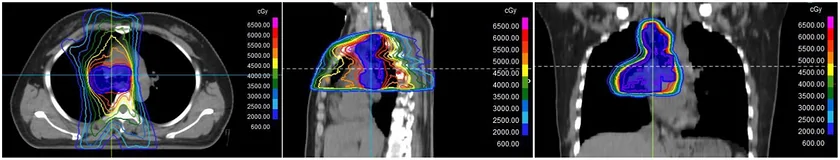
Dose distribution of a conventionally dose‐fractionated coplanar dual‐arc volume arc modulated radiotherapy (VMAT) plan for lung cancer.

Inverse planning is a manual trial‐and‐error process that is time‐consuming and easily influenced by the personal experience of dosimetrist, making it difficult for them to determine whether the final plan can be further improved. For the treatment plan of conventional fractional intensity‐modulated lung radiotherapy, there is a predictable functional relationship between the r and the MLD or V_20Gy_, with a better fit shown in formulas 3 and 4. Based on the r of the PTV volume on each patient's localized CT image to the contoured whole lung volume, the lowest possible MLD and V_20Gy_ can be predicted using the relationship between logarithmic and power functions. The MLD or V_20Gy_ as a function of r provides planners with tools to estimate MLD and V_20Gy_ in conventional dose fractionation plans. Because the *R* ^2^ values of the power function and logarithmic function are high, this paper accepts using the power function and logarithmic function to predict MLD and V20. The r is first calculated, then the MLD and V_20Gy_ are calculated using formulas 3 or 4, respectively. The results are input into the treatment planning system's optimization constraints for optimization calculations, and finally, the optimization values are adjusted based on the calculation results, and the lowest MLD and V_20Gy_ are obtained under scientific principles. By using the model in the article, the trial and error time is reduced by directly obtaining an approximate range of lowest lung dose. Moreover, the test set can also verify the effectiveness of the proposed model in optimizing planning and OAR. Figure 4 shows the dose distribution chart obtained from the treatment plan based on the predictive values derived from the r and the power and logarithmic function of the MLD or V_20Gy_. Furthermore, as shown in the residual plot of the fitting function, the relationship between MLD and V_20Gy_ has some outliers. These outliers may arise from factors other than the r, such as PTV shape, number of cross‐sections, and the position of risk structures. The longer the PTV in the left‐right direction (i.e., the flatter the shape) and the greater the extent of PTV in superior‐inferior direction, the higher the dose received by the lungs.

## 5 CONCLUSION

For conventional fractionated radiotherapy plans for lung cancer, the r is an important indicator for predicting lung dose. Planners and evaluators can use the power function or logarithmic function fitting relationships derived from this study to obtain predicted values for lung tumor plan evaluation metrics such as the MLD and V_20Gy_ in coplanar dual‐arc VMAT treatment plans. This helps to estimate the minimum dose range that can be delivered to the lung tissue, thereby enabling more appropriate lung cancer radiotherapy plan and reducing the incidence of radiation‐induced lung injury. This study only considered coplanar dual‐arc VMAT; results related to three‐dimensional conformal radiation therapy (3DCRT), intensity‐modulated radiation therapy (IMRT), non‐coplanar VMAT, tomotherapy, intensity‐modulated proton therapy (IMPT), and carbon ion therapy still need further investigation. Future work should consider other radiation therapy techniques and the function fitting relationship between MLD or V_20Gy_ and the r in conventional fractionated radiation therapy plans.

## AUTHOR CONTRIBUTIONS

Yu Wang collected and analyzed the data, performed statistical analyses, and drafted the manuscript. Deying Xu contributed to the clinical methodology and guided the manuscript revisions. Yunfeng Liu contributed to data collection and data analysis. Xiaofeng Zhang collected and analyzed the validation set data. Liang Yu supervised the study and revised the manuscript. All authors reviewed and approved the final manuscript.

## CONFLICT OF INTEREST STATEMENT

The authors declare no conflicts of interest.

## ETHICS STATEMENT

This study was approved by the ethics committee of Panjin Central Hospital. The requirement for written informed consent was waived, and all patients were given the opportunity to opt out.

## Data Availability

The original contributions presented in the study are included in the article/supplementary material. Further inquiries can be directed to the corresponding author.
